# Novel methodology for measuring intraoral wear in enamel and dental restorative materials

**DOI:** 10.1002/cre2.322

**Published:** 2020-09-21

**Authors:** Josephine F. Esquivel‐Upshaw, Shu‐Min Hsu, Ana C. Bohórquez, Nader Abdulhameed, Gary W. Scheiffele, Mijin Kim, Dan Neal, John Chai, Fan Ren

**Affiliations:** ^1^ Restorative Dental Sciences Division of Prosthodontics, University of Florida College of Dentistry Gainesville Florida USA; ^2^ Herbert Wertheim College of Engineering's Research Service Centers University of Florida College of Engineering Gainesville Florida USA; ^3^ Restorative Dentistry LECOM School of Dental Medicine Bradenton Florida USA; ^4^ Department of Neurosurgery University of Florida College of Medicine Gainesville Florida USA; ^5^ Department of Prosthodontics Northwestern University Dental School Chicago Illinois USA; ^6^ Department of Chemical Engineering University of Florida Herbert Wertheim College of Engineering Gainesville Florida USA

**Keywords:** monolithic zirconia, wear depth, wear of enamel, worn volume, X‐ray computed microtomography

## Abstract

**Objectives:**

To test the hypotheses that (a) the chairside/handheld dental scanner combined with a metrology software will measure clinical wear *in vivo* in agreement with measurements from X‐ray computed microtomography and; (b) polished monolithic zirconia does not cause accelerated wear of opposing enamel.

**Materials and methods:**

Thirty single crowns were randomized to receive a monolithic zirconia or metal‐ceramic crown. Two non‐restored opposing teeth in the same quadrants were identified to serve as enamel controls. After cementation, quadrants were scanned using an intraoral dental scanner. Patients were recalled at 6‐months and 1‐year for re‐scanning. Scanned images were compared using a metrology software to determine maximum vertical wear of teeth. The accuracy of the scanning measurements from this new method was compared with X‐ray computed microtomography (micro‐CT) measurements. Statistical analysis was performed using Mann–Whitney *U* test to determine significant differences between wear of enamel against zirconia, metal‐ceramic or enamel. Linear regression analysis determined agreement between measurements obtained using intraoral scanning and micro‐CT.

**Results:**

Regression analysis demonstrated that there is a quantitative agreement between depth and volume measurements produced using intraoral scanning and the micro‐CT methodologies. There was no significant difference between the wear of enamel against polished monolithic zirconia crowns and enamel against enamel.

**Conclusions:**

Intraoral scanning combined with a matching software can accurately quantify clinical wear to verify that monolithic zirconia exhibited comparable wear of enamel compared with metal‐ceramic crowns and control enamel. Agreement between the intraoral scanner and the micro‐CT was 99.8%. **Clinical Trials.gov** NCT02289781.

## 1 INTRODUCTION

Wear affects health in several ways, which include effects on supporting structures of the teeth, loss of vertical dimension of occlusion, tooth sensitivity, esthetics, and overall masticatory function (Oh, Delong, & Anusavice, 2002). Wear can lead to dysfunctions of the temporomandibular joint and the head with symptoms ranging from headache and pain to decreased function. Unfortunately, wear patterns, wear occurrence, and the amount of wear for any particular individual are specific to habit, diet, and musculature (Heintze, Cavalleri, Forjanic, Zellweger, & Rousson, 2008). A majority of wear studies were conducted *in vitro* using chewing simulators (Abe et al., 2001; Clelland, Agarwala, Knobloch, & Seghi, 2001; Clelland, Agarwala, Knobloch, & Seghi, 2003; Elmaria, Goldstein, Vijayaraghavan, Legeros, & Hittelman, 2006; Heintze, Cavalleri, Forjanic, Zellweger, & Rousson, 2006; Kadokawa, Suzuki, & Tanaka, 2006; Olivera & Marques, 2008) and these studies revealed no correlations with clinical occurrences in the mouth. Therefore, the mechanism of wear should be examined *in vivo* to fully assess the effects of different dental materials on the wear of enamel under individual conditions.

More recently, the assessment of *in vivo* wear has been conducted using laser scanner technology or profilometry with tooth replicates either in acrylic resin or stone (Heintze, Cavalleri, Forjanic, Zellweger, & Rousson, 2006; Mehl, Gloger, Kunzelmann, & Hickel, 1997; Pintado, Anderson, DeLong, & Douglas, 1997; Rodriguez & Bartlett, 2010; Rodriguez, Curtis, & Bartlett, 2009; Schlueter, Ganss, Sanctis, & Klimek, 2005). While this has proven to be effective, there are some errors involved in the replication process with the impression and the replicating materials (Hsu et al., 2019; Rodriguez & Bartlett, 2010; Segerström, Wiking‐Lima de Faria, Braian, Ameri, & Ahlgren, 2018). Also, the resolution accuracy of the laser scanners varies from 5 to 20 μm and can affect the quantification of wear. Depending on the type of laser beam wavelength used, errors can be introduced with the types and reflectance properties of the materials being scanned.

X‐ray microcomputed tomography (micro‐CT) is a nondestructive technique used to visualize the internal features and morphologic characteristics in detail (Fau et al., 2017; Kim et al., 2017; Kim, Paik, & Lee, 2007; Olejniczak & Grine, 2006; Swain & Xue, 2009; Van Oosterwyck, Duyck, Sloten, et al., 2000). This technology uses microfocal spot X‐ray as a source and has good spatial resolution with a range of 5–50 μm voxels (Swain & Xue, 2009). The qualitative and quantitative studies using micro‐CT have been reported (Kim et al., 2007; Kim et al., 2017; Olejniczak & Grine, 2006; Van Oosterwyck et al., 2000). A good correlation between micro‐CT images and histological sections was reported qualitatively (Van Oosterwyck et al., 2000). The quantitative results of digital dental restorations produced by micro‐CT demonstrated no significant difference in dimension compared with extraoral and intraoral optical methods (Kim et al., 2017). The difference detected during measurement of enamel thickness using micro‐CT images compared with physical sectioning was only 3 to 5% (Olejniczak & Grine, 2006). Micro‐CT has been considered the gold standard due to this instrument's high resolution and noninvasive measurement (Fau et al., 2017).

Computer Aided Design and Manufacturing (CAD‐CAM) technology has brought about the influx of intraoral scanners which enable scanning of tooth preparations for prosthetic fabrication (Logozzo et al., 2008; Trifković, Budak, Todorović, et al., 2012; Brandestini et al., 1989). For this study, we wanted to determine whether one of these intraoral scanners can be used in conjunction with a metrology software to quantify clinical wear on enamel opposing monolithic zirconia crowns. This procedure will essentially eliminate the replication required of the current methods for clinical wear quantification.

The purpose of this study was to test the hypotheses that: (a) the chairside/handheld dental scanner combined with a metrology software will measure clinical wear *in vivo* in agreement with measurements from X‐ray computed microtomography and; (b) monolithic zirconia does not cause accelerated wear of opposing enamel.

## 2 MATERIALS AND METHODS

### 2.1 Clinical study design

The protocol for this clinical study was previously reported (Esquivel‐Upshaw et al., 2018). A randomized, controlled, clinical trial was designed to analyze the wear of enamel by opposing polished monolithic zirconia crowns and by polished veneer surfaces of metal‐ceramic crowns. This single‐blind pilot study involved a total of 30 teeth that required full coverage crowns that oppose natural antagonist teeth. Institutional Review Board approval for treating human subjects using the research protocol was obtained. All participants were required to sign an informed consent form prior to initiating the study. Green stones were used to modify the shape of the crowns prior to polishing. Silicone polishers (Denerica Dental Corp, Saint Charles, IL) were used from coarse to fine grits for polishing both types of crowns. The final polish was attained using a diamond paste (DirectDia Paste Diamond Polishing Paste, Shofu Dental Corp) with a stiff bristle brush. The average polishing time for each crown was approximately 15 min. After crown cementation, wear on teeth opposing the crowns as well as control enamel teeth opposing one another were analyzed using the novel intraoral method for wear measurement. Baseline measurements of the crown were obtained after cementation and the participants were asked to return at 6 months and 1‐year for analysis of wear.

### 2.2 Wear quantification

This novel and direct method uses period scans obtained from the intraoral scanner (3M True Definition Chairside Oral Scanner Digital Impression System, 3M, ESPE) which were compared using a metrology software (Geomagic Control 2014, 3D systems) via stereolithography (STL) files. The accuracy of this scanner is reported to be less than 5 μm—or 0.3% for measured isotropic scale errors for a single tooth (Laboratories, 2018; Hack & Patzelt, 2015; Sevcik, Graham, Yun, Reff, & Deckard, 2014). When used for impression making, this scanner has a published fit rate of 99.7% for fabricated crowns (Laboratories, 2018; Hack & Patzelt, 2015; Sevcik et al., 2014). There was one clinician who performed all the intraoral scanning of the crowns, control enamel, and antagonists at 6 months and 1 year. Each scan was saved and uploaded to a computer equipped with the metrology software. For wear quantification using the metrology software, there was one lab operator who performed all superimposition and wear quantification procedures. Since there was a single operator for the intraoral scanning and wear quantification, there was no need to calibrate multiple operators. Both operators were properly trained on the procedures involved with intraoral scanning and use of the metrology software. The superimposition software is supposed to capture the best possible fit between the two period scans from 6 months and 1 year. To achieve this, the following were performed: (a) trimming of the soft tissue: prior to superimposition, each file was cropped to eliminate soft tissue to minimize the time of superimposition; (b) initial superimposition: the trimmed scan images were used for the initial superimposition. This was performed using the best fit alignment option to merge both period files together. This determined whether there was any over or under trimming of either period scan file; (c) trimming after initial superimposition: after the initial fit was performed, more trimming of the extraneous areas from the merged file was performed to facilitate a greater fit. The best fit alignment was applied repeatedly after each trimming; (d) isolating target teeth: the merged file was then duplicated and trimmed to leave only the target teeth for wear measurement; (e) trimming after isolating target teeth: this isolated merged file was magnified and re‐trimmed for more soft tissue or missing scanned data. The best fit option was once again applied and decreased to 0.02 mm and then 0.01 mm (10 μm). This means that this is the level that is detectable as a true signal so that any value above this threshold is real. After these procedures are applied, the best‐fit merged data is produced with a minimum amount of error for wear measurement between baseline, 6 months and 1 year. The maximum value for wear is 0.1 mm and the minimum value is −0.1 mm. These areas indicate critical borders and can indicate a mismatch or missing data between the scans. Also, the neutral areas indicate that there is minimum or no detectable change between the period scans. Intraoral photos taken with the occlusal contacts were used to further identify the plausible areas of wear.

### 2.3 Statistical analysis

All analyses were performed using the R statistical software package (V3.2.4, The R Foundation for Statistical Computing, Vienna, Austria). Since the sample size was small (*N* < 15 per group for all comparisons), the non‐parametric Mann–Whitney U test was used to compare wear between the Zirconia and metal‐ceramic crown types at 6 months and 1 year. To compare antagonist wear to control wear between the two groups, the difference between antagonist wear and control wear as the outcome for each patient was calculated. The mean wear of the two control teeth was used as the control wear for that patient. A regression analysis was performed to determine the agreement between the intraoral scanning and micro‐CT measurements using a simulated tooth.

### 2.4 Validity testing of intraoral scanner using computed microtomography (microCT)

A maxillary left first molar Dentoform® (Columbia Dentoform®) tooth was scanned using the micro‐CT (GE Phoenix v|tome|x m industrial scanner (GE's Inspection Technologies, LP, Pennsylvania) and the intraoral scanning technologies at baseline. The Dentoform® tooth was mounted and wear facets were created on different areas of the occlusal surface using a chewing simulator (CS Mechatronik GMBH). The Dentoform® tooth was opposed with a steatite ball under 49 N load for four different cycles, 25,000; 50,000; 100,000 and 120,000 with a speed of 60 mm/s and a horizontal movement of 0.7 mm. Wear facets were created in six different locations (A, B, C, D, E, F) on the same tooth to compare different wear facet depths. The tooth was scanned again using micro‐CT and intraoral scanning after inducing wear facets. Maximum depth and volume wear were analyzed based on the surface determination for Dentoform® tooth baseline and Dentoform® wear tooth samples using VGStudio Max 3.0 (Volume Graphics, Heidelberg, Germany). A metrology software was used to compare the stereolithography (STL) files derived from the intraoral scanner as described previously on the Dentoform® tooth.

### 2.5 X‐ray computed microtomography (micro‐CT) measurements and data processing

To investigate the volume and depth of the worn volumes, micro‐CT scanning was performed as a nondestructive testing by measuring a Dentoform® tooth at baseline and Dentoform® wear tooth samples. Scans were completed using the micro‐CT. Images were obtained using a 180‐kV X‐ray tube with a diamond target at a voltage of 100 kV and a current of 100 μA coupled with a beam collimator. The settings for image collection were the following: 250 ms detector time, average of three images with a skip of two images per rotation. This generated 1800 images and a voxel size of 10 μm. 2D projections were reconstructed using GE Phoenix Datos|x 2 reconstruction software version 2.4 (GE Sensing Inspection Technologies GmbH, Germany). Tomography slices were imported into VGStudio MAX 3.0 to perform volume analysis, 3D visualization and wall thickness analysis for worn areas.

### 2.6 X‐ray computed microtomography (microCT) calibration procedure and size validation

**FIGURE 1:**
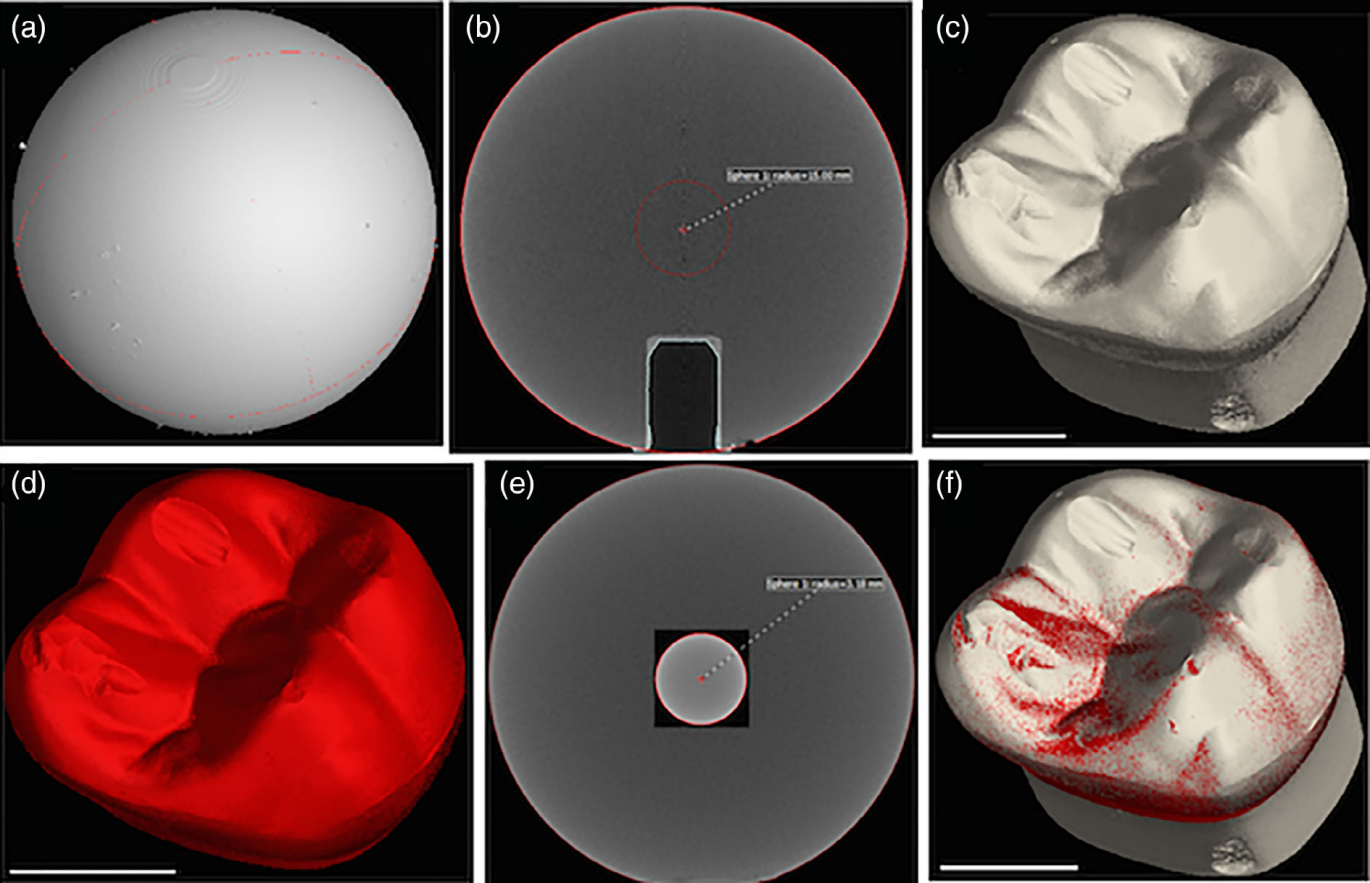
(a) 3D volume reconstruction of a 30‐mm ceramic sphere scanned using the same CT scan position selected for scanning the Dentoform® samples; (b) Front view of a tomography image showing as a sphere radius *r* = 15 mm; (c) Representative 3D visualization for the initial worn Dentoform® tooth scanned; (d) Representative 3D visualization for the worn Dentoform® tooth scanned for validation purposes; (e) Front view of a tomography image showing a silicon‐nitride ceramic ball having roughly comparable size and density as the Dentoform® samples; (f) Merging two surfaces obtained for initial scanned worn Dentoform® and rescanned worn Dentoform®. All reconstruction and segmentation analysis were made using VGStudio Max 3.0. Scale bar 5 mm (white line)

For geometric calibration, a 4‐point calibration was performed using GE Phoenix Datos|x 2 acquisition software (GE Sensing Inspection Technologies GmbH, Germany) employing a calibration rod mounted vertically in the sample manipulator. This was measured in two different Z‐axis positions and four different X‐axis positions that enclosed the expected scan location with a trapezoid focused upon the source of radiation. From these points the software calculates precise source to detector distance and X‐axis offsets. Then, a 30‐mm ceramic sphere type VTX18CQ000 (GE Sensing & Inspection Technologies GmbH, Germany) was scanned using the same CT scan position used for the Dentoform® samples as can be observed in Figure 1a, b. Hence, after calibration sphere data is segmented, visualized, and analyzed one can, if necessary, calculate a correction factor to use in our 3D volume data analysis software (VGStudio Max 3.0). The initial scans in this work were performed about 10 days after instrument calibration and approximately 2 days apart each sample (Dentoform® tooth baseline and worn Dentoform® tooth). A representative 3D visualization for the initial worn Dentoform® tooth can be seen in Figure 1c.

To validate our scan work, the worn Dentoform® tooth was rescanned, reconstructed and imported into VGStudio Max 3.0 (Figure 1d). This was done immediately after the above calibration procedure in which we obtained a calibration correction factor of 0.9925. In addition, we scanned a silicon‐nitride ceramic ball (9576K34, McMASTER‐CARR, Douglasville, GA) having roughly comparable size and density as the Dentoform® samples (Figure 1e). This ball was measured five times with a digital micrometer (CO 030025, Marathon Management Co) to find a diameter of 6,351 μm ± 2 μm. We used the same CT scan position for the Dentoform® samples and after applying the correction factor obtained as above, we obtained a value in close agreement with the diameter measured by the electronic caliper (6,360 μm). Finally, we merged two surfaces obtained for initial scanned worn Dentoform® and rescanned worn Dentoform® tooth using VGStudio Max 3.0 and the two surfaces were coincident as can be observed in Figure 1f. Under this approach, we were able to confirm the accuracy of the quantification of depth and worn volume in our study.

## 3 RESULTS

### 3.1 Clinical analysis

**FIGURE 2:**
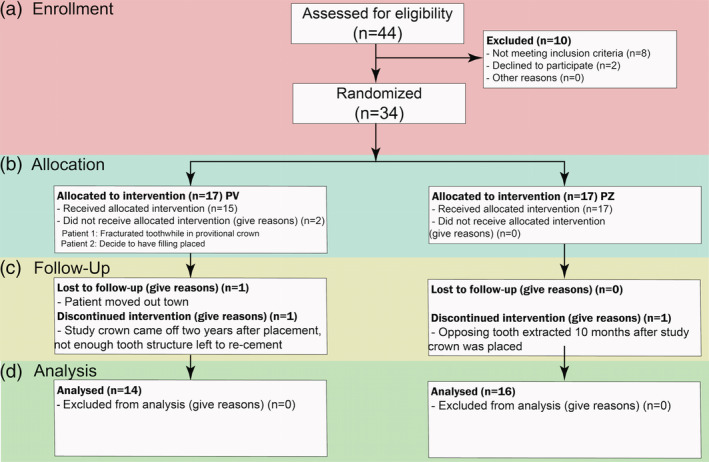
Consort diagram showing enrollment, allocation, follow up and analysis of participants

**FIGURE 3:**
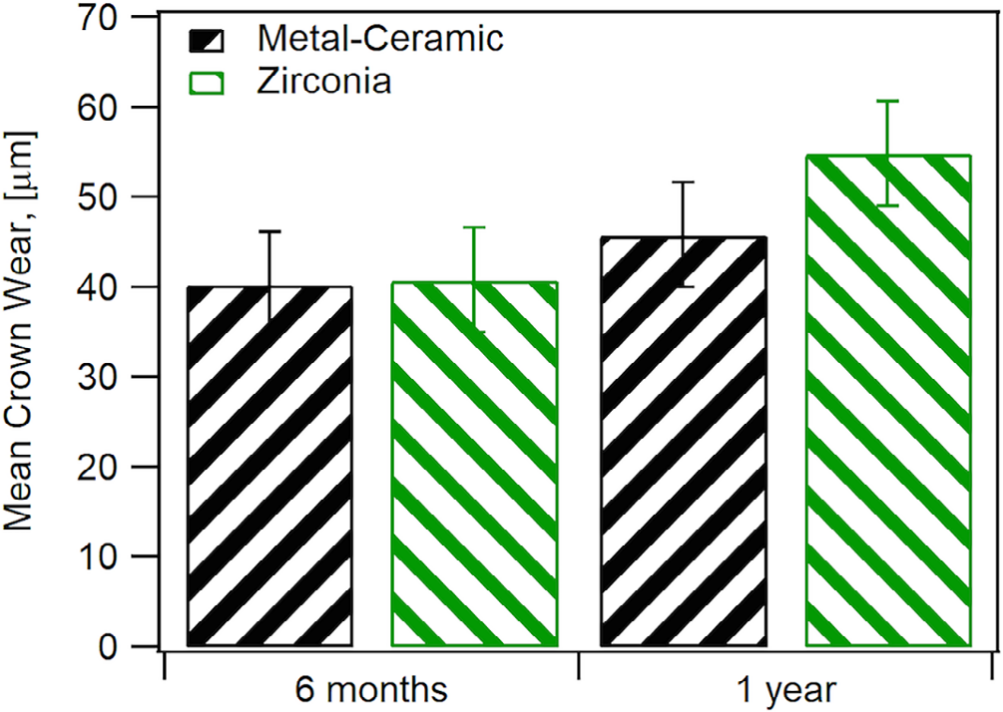
Comparison of wear between metal‐ceramic (MC) and monolithic zirconia crowns at 6 months and 1 year using two different methods

**FIGURE 4:**
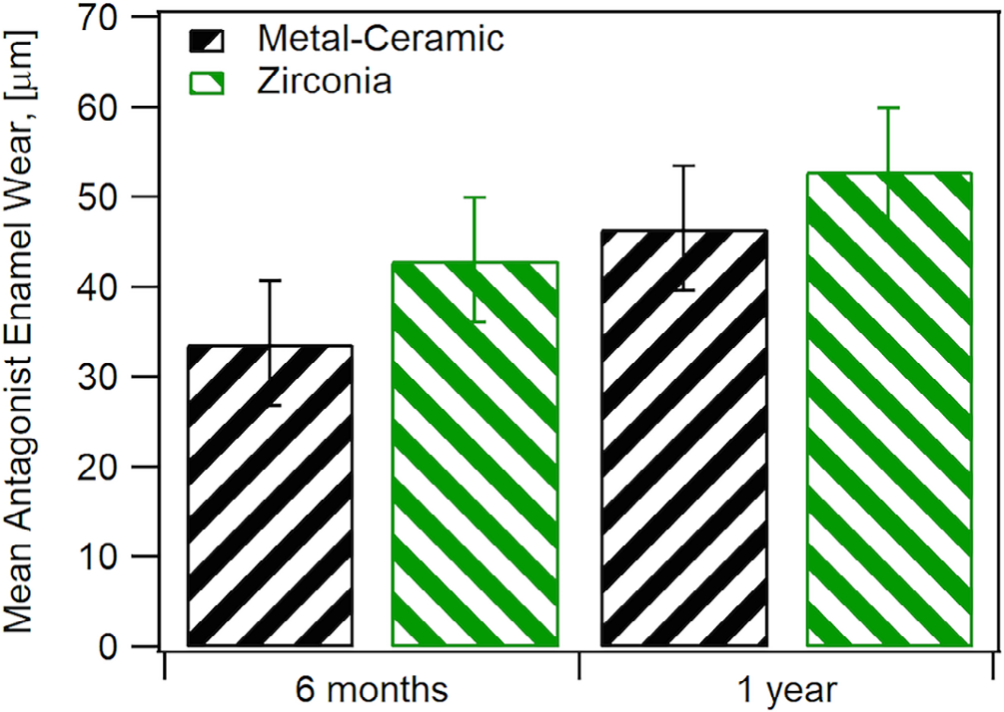
Comparison of antagonist enamel wear between metal ceramic and monolithic zirconia crowns at 6 months and 1 year using two different methods

**FIGURE 5:**
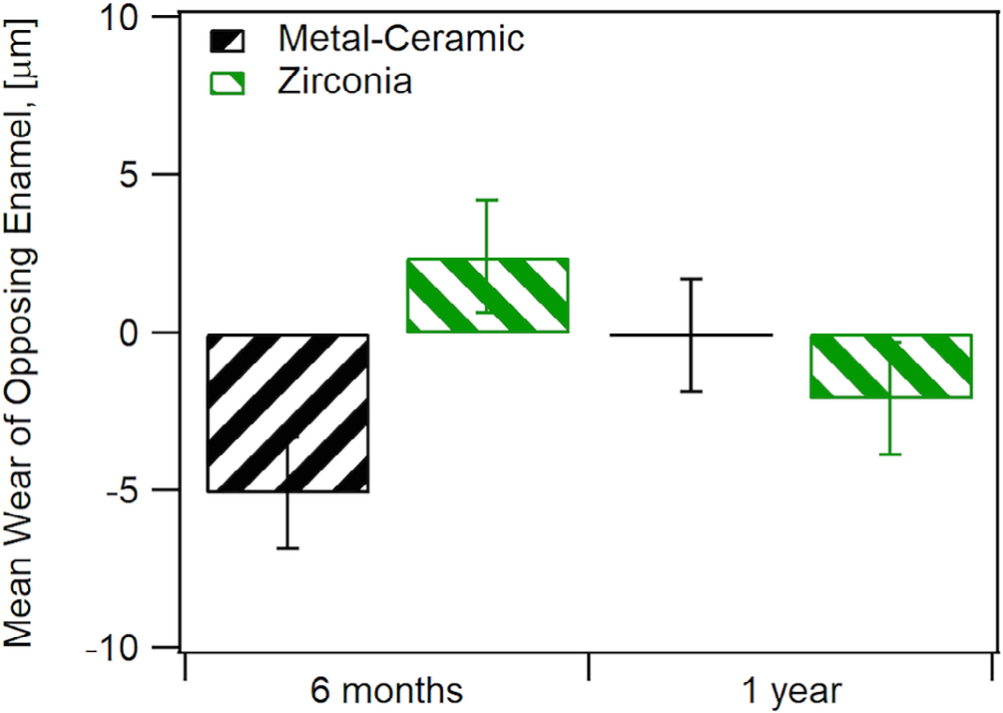
Comparison of enamel wear between crown antagonist enamel and control enamel. Negative values indicate greater control enamel wear while positive values indicate greater antagonist enamel wear

**FIGURE 6:**
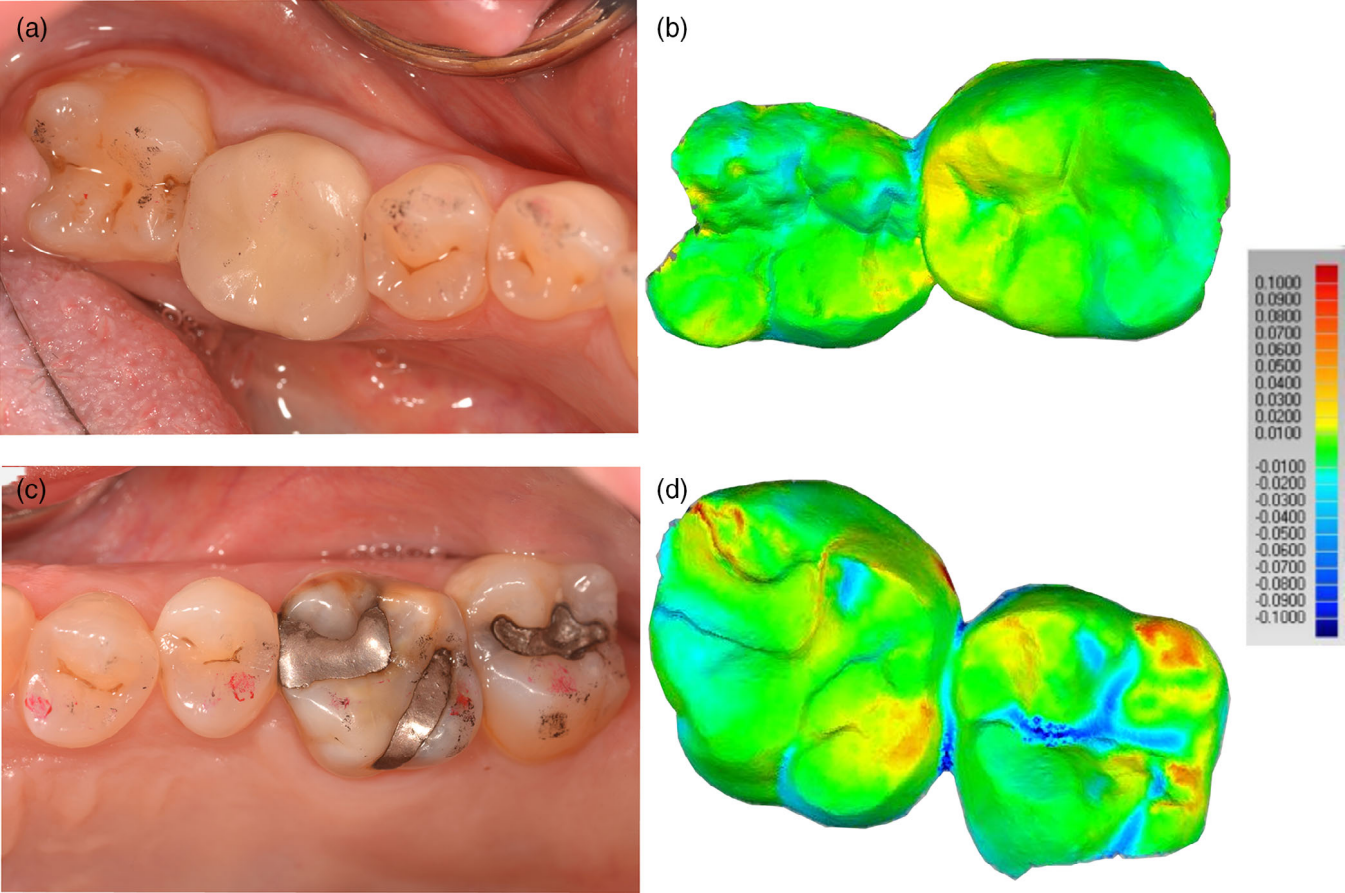
(a) Representative zirconia crown on mandibular left first molar, second molar was used as a control alongside scans using Geomagic® Control X™ software (3D Systems, Rock Hill, SC) where baseline scans are superimposed with either 6 months or 1‐year scans; (b) Opposing quadrant where maxillary first molar was used as the opposing enamel and the second molar was a control alongside intraoral scans. Scale indicate degrees of difference in microns between the two scans

Thirty teeth in twenty‐five enrolled participants (20 females, 5 males and no more than two crowns per participant), were included in this study and were seen from 2013–2017. There were 16 monolithic zirconia crowns (PZ) and 14 metal‐ceramic crowns (PV) analyzed. A more detailed distribution of participants can be seen in Figure 2 following the CONSORT diagram. Wear results for the monolithic zirconia and the opposing enamel using the indirect method where stone replicates were used were reported previously (Esquivel‐Upshaw et al., 2018). The results showed that there was no significant difference between the wear of enamel versus enamel, enamel versus metal‐ceramic and enamel versus monolithic zirconia. A comparison of the same cohort using the intraoral scanning technology for measuring wear between the monolithic zirconia and metal‐ceramic crowns were compared at 6 months and 1 year. As shown in Figure 3, no significant differences were observed at any time point (6 months *p* = 1; 1‐year *p* = .165) using intraoral scanning. The wear of the enamel opposing both types of crowns was also compared to determine if one material wore the opposing enamel more than the other as shown in Figure 4. There were no significant differences observed for antagonist enamel wear across all time periods (6 months *p* = .152; 1‐year *p* = .235). The opposing enamel wear was then compared to the wear between two opposing enamel surfaces (control wear) to determine if either material caused an increase in opposing enamel wear. This was computed for by the difference between antagonist wear and control wear for each participant. The mean between the two controls were subtracted from the antagonist enamel wear of the crowns. In Figure 5, negative numbers indicate more control wear than antagonist wear and positive values indicate more antagonist tooth wear. The *p* values are *p* = .034 for 6 months and *p* = .843 for 1‐year. The enamel opposing the zirconia crown showed greater wear than the enamel opposing the metal ceramic when compared with their controls for the first 6 months. However, this difference seems to even out by the first year. Figure 6 shows the metrology software results comparing two representative scans performed by using intraoral scanning. A clinical image from an actual patient is shown (Figure 6a) with the zirconia crown on the left mandibular first molar. Figure 6b shows the superimposed period scans from baseline to either 6 months or 1 year. The opposing quadrant is shown (Figure 6c) with the left maxillary first molar as the antagonist enamel tooth and the opposing second molars as the control enamel teeth. Figure 6d is the superimposed images of the opposing quadrant.

### 3.2 Validity measurement with micro‐CT

**FIGURE 7:**
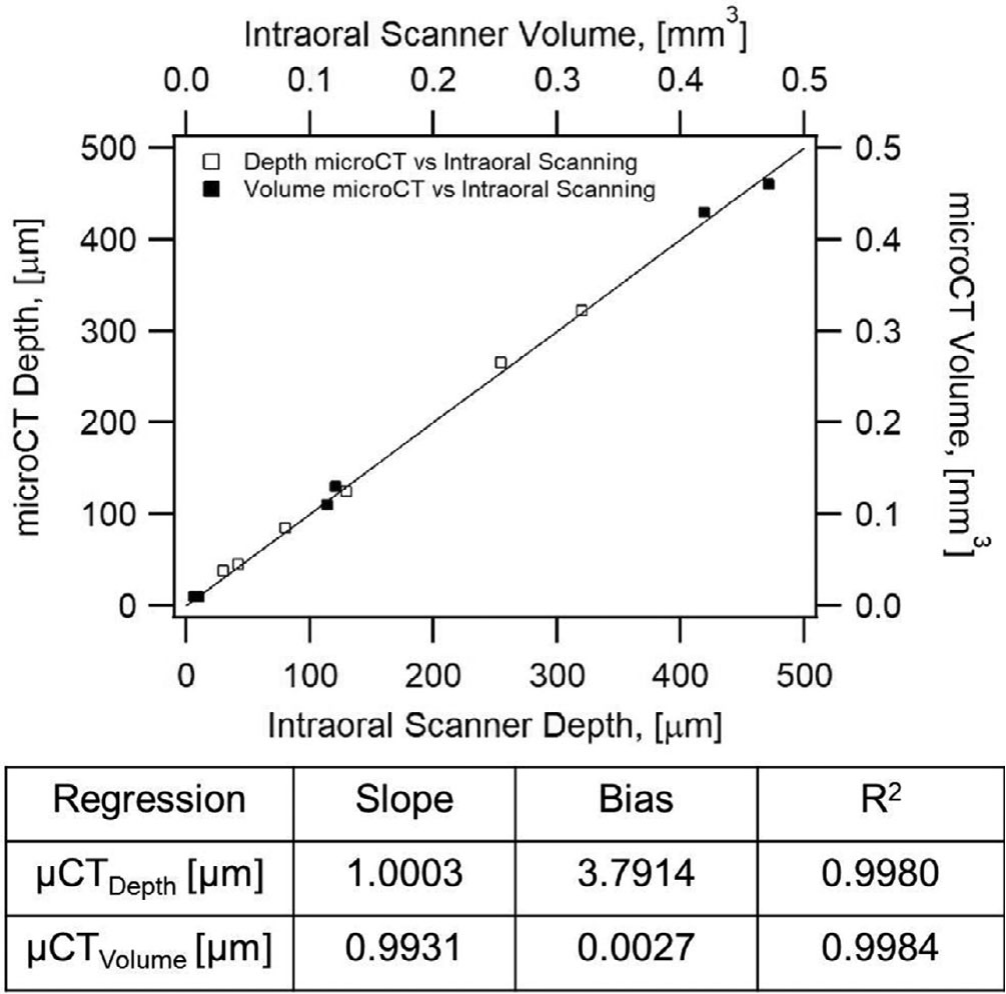
Regression analysis showing intraoral Parity plot for depth (open squares) and volume (closed squares) of worn areas obtained from micro‐CT and intraoral scanning. Agreement for depth is 99.8% and 99.84 for volume

**FIGURE 8:**
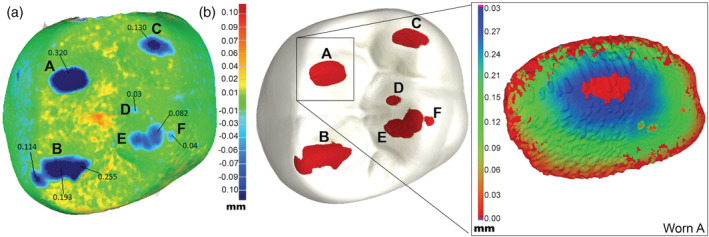
(a) Top view of superimposed baseline scan and worn scan from intraoral scanner using metrology software. Blue areas show areas of wear according to color scale on the right of the image; (b) Top view of a Dentoform model showing wear facets (red) obtained using micro‐CT measurements and representative wall thickness analysis obtained using VGStudio Max 3.0 for worn area A

Regression analysis shows that dental wear values estimated by intraoral scanning employing the metrology software as a tool agree with the depth and volume wear quantification based on micro‐CT analysis using surface determinations and wall thickness analysis procedures in VGStudio Max 3.0. Good quantitative agreement was observed when comparing depth and volume measurements obtained from micro‐CT and intraoral scanning approaches, which is evident in Figure 7. Worn areas obtained by intraoral scanning and micro‐CT measurements are shown in Figure 8. Figure 8a illustrates the intraoral scanning representation with superimposed images from baseline and 1 year while Figure 8b shows a representative wall thickness analysis result for the area labeled “Worn A."

## 4 DISCUSSION

Clinical studies have confirmed that polished monolithic zirconia demonstrate comparable wear with enamel wear controls (Cardelli, Manobianco, Serafini, Murmura, & Beuer, 2016; Esquivel‐Upshaw et al., 2018; Lohbauer & Reich, 2016; Mundhe, Jain, Pruthi, & Shah, 2015; Stober, Bermejo, Schwindling, & Schmitter, 2016). Studies have shown that polished zirconia is recommended over glazed zirconia with the wear of antagonist enamel measuring less for polished zirconia versus glazed (Janyavula et al., 2013; Lawson, Janyavula, Syklawer, McLaren, & Burgess, 2014; Rupawala et al., 2017). The reason for this is that the glaze material tends to wear off, exposing the rougher zirconia surface underneath to cause increased wear. In addition, the glaze particles act as a third‐body abrasive and produce a rougher surface. The results in this study indicate that the wear of enamel opposing zirconia was higher than the control wear at 6 months but then no difference is detected between zirconia and metal‐ceramic after 1 year. These results are in agreement with Stober's (Stober et al., 2016) findings at 6 months, 1 year, and 2 years, where an initial wear increase occurred at 6 months prior to reaching a plateau towards 2 years. In addition to the surface roughness and material properties, differences in wear can be attributed to patient's saliva, occlusion, and dietary habits.

Analysis of wear results from this clinical study using an indirect technique with stone replicates was reported in a previous publication (Esquivel‐Upshaw et al., 2018). This current study utilized a direct method for measuring clinical wear where previous clinical studies used replicates of the teeth in either stone or acrylic to facilitate wear measurement (Cardelli et al., 2016; Lohbauer & Reich, 2016; Mundhe et al., 2015; Stober et al., 2016). This novel and direct method employs an intraoral scanner originally used to scan tooth preparations for CAD‐CAM crown fabrication. The advantage of direct measurement of the teeth is that errors resulting from the replication process are minimized. Results for 6 months and 1‐year wear confirm the viability of monolithic zirconia as a restorative material using this direct method for wear measurement.

Variations inherent to the direct method include the amount of plaque, debris and saliva present on the teeth prior to scanning. Use of this technique mandates that the scanned teeth are free of extraneous materials which could produce erroneous readings. Additionally, teeth should be sprayed with zinc oxide powder to minimize the reflectance of the surface and promote better scanning (Logozzo et al., 2008; Trifković et al., 2012). Statistically, this direct method produced well‐behaved normal distributions and the conclusion that monolithic zirconia does not unduly wear opposing enamel coincides with the results from the previous technique which utilized stone replicates.

The viability of this direct method was further verified using micro‐CT measurements. The accuracy of micro‐CT scanning as a nondestructive and metrological tool has been widely documented in various fields of study (Kline, Zamir, & Ritman, 2010; Pelletti et al., 2017). A calibration and validation of the micro‐CT was performed to determine the accuracy of this technique in a Dentoform® tooth baseline and worn Dentoform® tooth. Based on measurements of the size of calibration spheres, an error of 0.15% was detected when the diameter of a calibration sphere of 30 mm was measured. This indicates that the microCT has a threshold error of approximately 10 μm with an object size of about 7 mm (teeth). The micro‐CT measurements were used to analyze accurate volumetric loss as well as maximum depth of the worn facets. Both intraoral scanning and the micro‐CT technologies identified the same areas of wear and demonstrated similar values for maximum depth loss and volume of the worn area. The maximum depth and volume values for worn areas positioned along the 45° diagonal in the parity plot shown in Figure 7 indicate that the predictions for wear depth and volume values by micro‐CT measurements and intraoral scanning correlate with each other. Regression analysis for depth confirmed that micro‐CT and intraoral scanner registered almost indentical values and that the wear measured by micro‐CT for any point is expected to be only 0.03% higher than that of the intraoral scanner. There is a line bias of 3.79 μm which translates to Equation (1)

(1) $$\mathrm{\mu C}T_{\text{Depth}}\left[\mathrm{\mu m}\right]=1.0003∙\text{intraora}l_{\text{Depth}}\left[\mathrm{\mu m}\right]+3.79$$

where,

*μCT*_*Depth*_[*μm*] is the micro‐CT depth wear in microns and *intraoral*_*Depth*_[*μm*] is the intraoral scanner depth wear.

The line bias would seem irrelevant since the threshold for error for both devices is 10 μm as explained earlier. The regression analysis for volume confirms this agreement with R^2^ = 99.84%, a line slope of 99% (micro‐CT registers 1% lower for any point) and a line bias of 0.002 μm. Therefore, both the micro‐CT and intraoral scanner produced the same wear values consistently for both depth and volume measurements. The more critical parameter in wear measurement is the maximum depth loss since occlusal contact areas can be broad and register high volumetric loss but not really have any indication on the adverse wear potential of the material.

The intraoral scanner used in this study operates on a blue light beam which has a shorter wavelength of 450 nm. Shorter wavelength beams have more resolution, less diffraction, and have more writing density (OpenStax, 2015). As a result of this, they penetrate less into the material being measured and cause less unfocused areas. Blue light scanning is indicated for organic, shiny, translucent, or transparent materials since less light diffusion occurs and a more accurate scan is generated. Therefore, blue light scanners, such as the intraoral scanner reported in this work, are ideal for scanning teeth intraorally.

In summary, this study demonstrated a novel method for measuring clinical wear on teeth (crowns and enamel) without the need to use replicates in either stone or acrylic. The method concurs with results published previously which utilized an indirect method of measuring the wear of zirconia on enamel. The validity of the measuring technique was also verified by X‐ray computerized microtomography. This new method confirms that zirconia does not cause accelerated wear of the opposing enamel.

## 5 CONCLUSION

This study demonstrates that the novel and direct method of wear measurement where an intraoral scanning device was used in conjunction with a 3D matching software is an acceptable method for measuring wear. The accuracy of this technique was compared with micro‐CT technology and wear values demonstrated quantitative agreement of 99.8% between the two methods. Measurements using this technique further confirm that polished monolithic zirconia does not cause accelerated wear of the opposing enamel. The wear of both metal‐ceramic and monolithic zirconia is comparable and that there are no significant differences noted between the enamel antagonist wear and control enamel wear of the two materials.
